# Characterization of the complete chloroplast genome sequence of *Koelreuteria bipinnata*

**DOI:** 10.1080/23802359.2020.1806129

**Published:** 2020-09-08

**Authors:** Yunzhou Lyu, Xiaoyun Dong, Hainan Sun, Libin Huang

**Affiliations:** Jiangsu Academy of Forestry, Nanjing, China

**Keywords:** *Koelreuteria bipinnata*, chloroplast genome, phylogenetic analysis

## Abstract

*Koelreuteria bipinnata* is an important ornamental tree with attractive flowers and fruits. In this study, we used next-generation sequencing technology to obtain the complete chloroplast genome of *K. bipinnata*. The entire genome was determined to be 163,863 bp in size, harboring a typical quadripartite structure with a large single copy (LSC) region of 90,240 bp, a small single copy (SSC) region of 18,883 bp, and a pair of 27,370 bp inverted repeat (IR) regions. The genome was predicted to contain 132 genes, including 84 protein-coding genes, 40 tRNA genes, and 8 rRNA genes. The overall GC content of *K. bipinnata* chloroplast genome was 37.29%. Phylogenetic analysis based on complete chloroplast genome sequences indicated that *K. bipinnata* was closely related to *K. paniculate*. This study would be useful for future population genetics studies and phylogenetic analysis of *K. bipinnata*.

*Koelreuteria bipinnata*, also named *K. bipinnata* Franch. var. integrifoliola (Merr.) T. Chen, is an extremely valuable tree species in Sapindaceae family (Feng, Li, et al. 2009). Traditionally, its dry flowers were used as herbal medicine for curing eye disease, and woods were utilized for furniture production in China. Nowadays, it has become a popular tree for urban landscaping as its attractive flowers and fruits (Lyu et al. 2017). Previously, the studies regarding *K. bipinnata* were mainly focus on its tissue culture (Feng, Li, et al. 2009; Feng, Zhang, et al. 2009; Cao et al. 2018), few data are currently available about its genomic information. In plants, the chloroplast genome is helpful for population genetics study, species-level identification, and phylogenetic analysis (Liu et al. 2020; Mo et al. 2020). Here, we *de novo* assembled the complete chloroplast genome of *K. bipinnata*, and analyzed its relationship with closely related species.

Leave samples of *K. bipinnata* was obtained from the experimental farm of Jiangsu Academy of Forestry (Nanjing, China 118°45′57.30″E, 31°51′27.94″N) and the voucher specimen was stored at Herbarium of Jiangsu Academy of Forestry (JAF; voucher: Lyu20200512-1). Total genomic DNA was extracted using Tiangen Plant Genomic DNA Kit (Tiangen Biotech Co., Beijing, China). The obtained DNA was fragmented to construct a paired-end library with an insert-size of 300 bp, and then the library was sequenced on the Illumina NovaSeq system (Illumina, San Diego, CA, USA). Following sequencing, 5.59 Gb of raw data were generated. Raw reads were processed by the Trimmomatic v0.32. *De novo* assembly and annotation of *K. bipinnata* chloroplast genome was performed by NOVOPlasty and DOGMA, respectively. The annotated cp genome was deposited in GenBank (Accession number: MT675915).

The whole chloroplast genome of *K. bipinnata* was 163,863 bp in size, and presented a typical quadripartite structure with a large single copy (LSC) region of 90,240 bp, a small single copy (SSC) region of 18,883 bp, and two separated inverted repeats (IRa and IRb) of 27,370 bp each. A total of 132 genes were predicted to include in the chloroplast genome: 84 protein-coding genes, 40 tRNA genes, and 8 rRNA genes. Of these genes, 7 protein-coding genes, 8 tRNA genes, and 4 rRNA genes have two copies, one in each of the IR regions. The overall GC content of the *K. bipinnata* chloroplast genome was 37.29%.

Phylogenetic tree was constructed using the chloroplast genome sequences of 21 representative Sapindales species: 12 from Aceraceae, 1 from Hippocastanaceae, and 8 from Sapindaceae. Multiple sequence alignment was completed with MAFFT (Katoh and Standley 2013) and then phylogenetic analysis was constructed using maximum-likelihood method implemented in IQ-tree software (Nguyen et al. 2015). The results showed that the clades in the phylogenetic tree have high bootstrap value, and species belonging to the same family were grouped together. Additionally, a close relationship was founded between *K. bipinnata* and *K. paniculate*. The complete chloroplast genome of *K. bipinnata* would be useful for its phylogenetic position determination as well as future population genomic studies (Figure 1).

**Figure 1.**
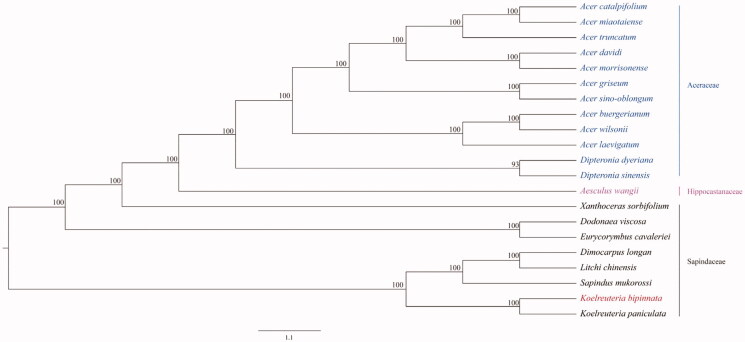
Phylogenetic tree constructed using ML method based on the complete chloroplast genome sequences of *K. bipinnata* and other 20 species. The bootstrap support values were shown at the branches. The accession numbers are the following: *Acer amplum*, NC_041080; *Acer buergerianum*, KF753631; *Acer davidii*, KU977442; *Acer griseum*, NC_034346; *Acer laevigatum*, NC_042443; *Acer miaotaiense*, KX098452; *Acer morrisonense*, KT970611; *Acer sino-oblongum*, NC_040106; *Acer truncatum*, NC_037211; *Acer wilsonii*, NC_040988; *Aesculus wangii*, NC_035955; *Dimocarpus longan*, NC_037447; *Dipteronia dyeriana*, KT985457; *Dipteronia sinensis*, KT878501; *Koelreuteria paniculata*, NC_037176.1; *Sapindus mukorossi*, KM454982; *Xanthoceras sorbifolium*, NC_037448; *Dodonaea viscosa*, NC_036099.1; *Eurycorymbus cavaleriei*, NC_037443; *Litchi chinensis*, NC_035238.

## Data Availability

The data that support the findings of this study are openly available in NCBI at http://www.ncbi.nlm.nih.gov/, reference number MT675915.

## References

[CIT0001] Cao L, Liu J, Lin Q, De Craene LPR. 2018. The floral organogenesis of *Koelreuteria bipinnata* and its variety *K*. *bipinnata* var. integrifolia (Sapindaceae): evidence of floral constraints on the evolution of monosymmetry. Plant Syst Evol. 304(8):923–935.

[CIT0002] Feng D, Li W, Zhang J, Li P, Li M, Zhao S, Shi B. 2009. Observation on somatic embryogenesis and histology in *Koelreuteria bipinnata* var. integrifoliola. Plant Physiol Commun. 45(9):855–858.

[CIT0003] Feng DL, Zhang J, Liu X, Peng WX, Wu TY. 2009. *In vitro* culture of immature embryos from *Koelreuteria bipinnata* var. integrifoliola. For Stud China. 11(3):179–184.

[CIT0004] Katoh K, Standley DM. 2013. MAFFT multiple sequence alignment software version 7: improvements in performance and usability. Mol Biol Evol. 30(4):772–780. 23329690 10.1093/molbev/mst010 PMC3603318

[CIT0005] Liu J, Chen T, Zhang Y, Li Y, Gong J, Yi Y. 2020. The complete chloroplast genome of *Rhododendron delavayi* (Ericaceae). Mitochondr DNA Part B. 5(1):37–38. 10.1080/23802359.2019.1689860 PMC7721020 33366411

[CIT0006] Lyu Y, Dong X, Huang L, Huang L. 2017. *De novo* assembly of *Koelreuteria* transcriptome and analysis of major gene related to leaf etiolation. S Afr J Bot. 113:355–361.

[CIT0007] Mo Z, Lou W, Chen Y, Jia X, Zhai M, Guo Z, Xuan J. 2020. The chloroplast genome of *Carya illinoinensis*: genome structure, adaptive evolution, and phylogenetic analysis. Forests. 11(2):207.

[CIT0008] Nguyen LT, Schmidt HA, Von Haeseler A, Minh BQ. 2015. IQ-TREE: a fast and effective stochastic algorithm for estimating maximum-likelihood phylogenies. Mol Biol Evol. 32(1):268–274. 25371430 10.1093/molbev/msu300 PMC4271533

